# A Role for PPAR*β*/*δ* in Ocular Angiogenesis

**DOI:** 10.1155/2008/825970

**Published:** 2008-03-11

**Authors:** David Bishop-Bailey

**Affiliations:** ^1^Centre of Translational Medicine and Therapeutics, William Harvey Research Institute, Barts and The London, Queen Mary's School of Medicine and Dentistry, Charterhouse Square, London EC1M 6BQ, UK

## Abstract

The uses of highly selective PPAR*β*/*δ* ligands and PPAR*β*/*δ* knockout mice have shown a direct ability of PPAR*β*/*δ* to regulate angiogenesis in vitro and in vivo in animal models. PPAR*β*/*δ* ligands induce the proangiogenic growth factor VEGF in many cells and tissues, though its actions in the eye are not known. However, virtually, all tissue components of the eye express PPAR*β*/*δ*. Both angiogenesis and in particular VEGF are not only critical for the development of the retina, but they are also a central component in many common pathologies of the eye, including diabetic retinopathy and age-related macular degeneration, the most common causes of blindness in the Western world. This review, therefore, will discuss the recent evidence of PPAR*β*/*δ*-mediated angiogenesis and VEGF release in the context of ocular disorders.

## 1. INTRODUCTION

Peroxisome proliferator-activated receptors (PPAR's) belong to the steroid receptor superfamily of ligand-activated transcription factors [1]. Three PPAR's, PPAR*α*, PPAR*β*/*δ*, and PPAR*γ*, have been identified [2]. PPAR*α* is predominantly expressed in liver, heart, kidney, brown adipose tissue, and stomach mucosa; PPAR*γ* is found primarily in adipose tissue; PPAR*β*/*δ* is the most ubiquitously expressed [3, 4], though its roles in physiological and pathophysiological processes are far from clear, particularly, in human tissue. The recent development of PPAR*β*/*δ* knockout and transgenic mice has started to implicate roles for PPAR*β*/*δ* in adipose tissue formation, metabolism, wound healing, brain development, placental function, atherosclerosis, colorectal carcinogenesis, and skeletal muscle function [5–7]. In this review, the emerging role of PPAR*β*/*δ* in regulating endothelial function and angiogenesis will be discussed with a particular emphasis to its relevance in the eye.

## 2. PPAR*β*/*δ* LIGANDS

A number of synthetic PPAR*β*/*δ* compounds have been described including GW0742X, GW2433, GW9578, L-783,483, GW501516, L-796,449, L-165,461, and compound F [8, 9]. In addition, putative endogenous PPAR*β*/*δ* activators include fatty acids [3, 10], triglycerides [11], the cyclooxygenase (COX) product, prostacyclin [10], the COX/prostacyclin synthase derived endocannabinoid metabolites [12], and *all-trans* retinoic acid (ATRA) [13]. ATRA is derived from vitamin A (retinol) which is found at its highest levels in the eye and is essential for its development and function [14]. Retinol is converted to retinaldehyde, a component of rhodopsin [14] and a functional PPAR*γ* antagonist [15, 16], which in turn is metabolised to ATRA by retinal dehydrogenases [14]. ATRA has its own family of high-affinity nuclear receptors, the retinoic acid receptor (RAR)*α*, -*β*, and -*γ*, which like the PPAR's act as heterodimers with RXR*α*, -*β*, and -*γ*, the receptors for the ATRA isomer 9-cis retinoic acid [17]. Although ATRA can activate PPAR*β*/*δ*, it is not known which, if any, of its actions are mediated by PPAR*β*/*δ*. However, since ATRA is present in such large quantities in ocular tissue, it is potentially an important site for its actions.

## 3. PPAR*β*/*δ* AND ENDOTHELIAL CELLS

Endothelial cells play critical roles in vascular biology, being both the protective inner lining of vessels and the local site for delivery of oxygen to all tissues. Angiogenesis is the process of new blood vessel/capillary formation from existing vessels, and hypoxia is a major signal which drives the process [18]. PPAR*α*, PPAR*β*/*δ*, and PPAR*γ* are all expressed in endothelial cells [19]. PPAR*α* and PPAR*γ* have well-characterised roles in endothelial cells, both being in general anti-inflammatory, antiproliferative [1], and antiangiogenic in a variety of in vitro and in vivo models, including tumorigenesis [20] and laser-induced retinal injury [21]. In contrast, the role of PPAR*β*/*δ* in this important cell type has only recent starting to be elucidated. Initial reports using prostacyclin as a ligand suggested that like PPAR*α* and PPAR*γ*, PPAR*β*/*δ* promoted endothelial cell apoptosis [22]. In contrast, the use of highly selective synthetic ligands has revealed a contradictory role for PPAR*β*/*δ* regulating endothelial cell survival, proliferation, and angiogenesis.

### 3.1. PPAR*β*/*δ* and endothelial cell proliferation and survival

Long- [23] and short-term [24] culture of endothelial cells with the selective ligand GW501516 induces endothelial cell proliferation, an effect associated with the induction of the VEGF receptor (Flt-1; VEGF R1) and VEGF production [23, 24]. In addition to inducing proliferation, PPAR*β*/*δ* activation protects cells from oxidant-induced apoptosis. Synthetic PPAR*β*/*δ* ligands or activation of the COX-prostacyclin pathway, which signals through PPAR*β*/*δ*, induce the endothelial expression of 14-3-3*α* protein [25]. 14-3-3 proteins are antiapoptotic and anti-inflammatory molecules [26]. PPAR*β*/*δ*-induced 14-3-3*α* blocks oxidant- (H_2_O_2_-) induced apoptosis by sequestering the proapoptotic protein Bad, stopping its translocation to mitochondrial membranes, where it initiates cytochrome c release and the subsequent activation of the proapoptotic caspase cascade [25].

### 3.2. PPAR*β*/*δ* and angiogenesis

In addition to having effects on endothelial cell proliferation, PPAR*β*/*δ* activation potently induces angiogenesis of human vascular endothelial cells in tumour extracellular matrix in vitro and in a murine matrigel plug model in vivo [24]. In addition, the putative PPAR*β*/*δ* ligand prostacyclin analogues [27] and ATRA [28] also induce angiogenesis, though the latter appears mostly dependent on its RAR*α* receptor rather than PPAR*β*/*δ* [29]. In human endothelial cells, a major trigger for morphogensis induced by PPAR*β*/*δ* stimulation was the stimulated release of VEGF [24]. In addition to VEGF, mRNA for the matrix metalloproteinase (MMP)-9, a protease important for cell migration was also elevated by PPAR*β*/*δ* activation [24]; however, whether this was secondary to VEGF release was not tested. VEGF is expressed as four main splice variants (by amino acid size: VEGF_121_, VEGF_165_, VEGF_189_, VEGF_206_) [29]. VEGF (VEGF-A; VEGF_165_) is a well-characterised central mediator of endothelial cell growth and angiogenesis [29, 30]. Two endothelial VEGF tyrosine kinase receptors have been identified: VEGFR-1/Flt-1, and VEGFR-2/KDR/Flk1. VEGF R2 appears to be the most important receptor in VEGF-induced mitogenesis and permeability [29, 30]. In addition, in two recent studies, the growth of PPAR*β*/*δ* wild-type tumours or angiogenesis in matrigel plugs in PPAR*β*/*δ* knockout mice was tested [31, 32]. The tumours in PPAR*β*/*δ* knockout mice compared to wild-type mice were associated with a diminished blood flow and an immature hyperplastic microvascular structures. Moreover, the retroviral introduction of PPAR*β*/*δ* into matrigel plugs was able to rescue the knockout phenotype by triggering microvessel maturation [31]. In the latter of these studies, PPAR*β*/*δ* was examined in tumours from patients who had undergone “angiogenic switch” a proangiogenic state involved in tumour progression [32]. PPAR*β*/*δ* correlated with advanced pathological tumor stage, increased risk for tumor recurrence, and distant metastasis, and was, therefore, suggested as a hub node transcription factor regulating tumour angiogenesis [32].

Genomic and proteomic analyses of the PPAR*β*/*δ* knockout endothelial cells isolated from matrigel plugs have also led to the identification of a number of additional candidate genes to mediate the actions of PPAR*β*/*δ* in angiogenesis. In particular, the Cdkn1c gene which encodes the cell cycle inhibitor p57^Kip2^ is a direct PPAR*β*/*δ* target gene that mediates PPAR*β*/*δ* effects on cell morphogenesis [31]. In addition, CD36 and thrombospondin were also decreased in matrigel-invading endothelial cells from PPAR*β*/*δ* knockout mice [31]. Thrombospondins by directly interacting with CD36 inhibit angiogenesis in vivo [33, 34]. Similarly, a proteomic analysis by the same group [35] on PPAR*β*/*δ* knockout endothelial cells has also revealed a decreased expression of the chloride intracellular channel protein (CLIC)-4 in migrating endothelial cells from PPAR*β*/*δ* knockout mice. In contrast, the expression of cellular retinol binding protein CRBP1 is increased in migrating endothelial cells from PPAR*β*/*δ* knockout mice [35]. CLIC-4 promotes and plays an essential role during tubular morphogenesis [36], while CRBP1 inhibits cell survival pathways by acting as an inhibitor of the AKT signalling pathway [37], an additional important signalling signal for angiogenesis to occur [38].

The combination of these studies show PPAR*β*/*δ* activation induces endothelial cell mitogen and differentiation signals, including VEGF, 14-3-3*α*, CD36 and thrombospondin, *CLIC4*, CRBP-1, and p57^KIP2^, all of which may act in a coordinate manner to bring about the functional morphogenic changes associated with angiogenesis.

### 3.3. PPAR*β*/*δ* and VEGF

Although the direct evidence for a role of PPAR*β*/*δ* in angiogenesis is relatively new, there has been an increasing literature regarding PPAR*β*/*δ* regulated tumour cell growth via inducing tumour cells to release VEGF. PPAR*β*/*δ* ligands induce VEGF in bladder cancer cells [39], human breast (T47D, MCF7), and prostate (LNCaP, PNT1A) cancer cell lines, along with its receptor flt-1 [22], but not (HT29, colon; HCT116, colon; LS-174T, colon; HepG2, hepatoma; and HuH7, hepatoma) cell lines [40].

In a genetic model of intestinal polyp development APC/min mouse, deletion of PPAR*β*/*δ* decreases intestinal adenoma growth and inhibits tumour-promoting effects of the PPAR*β*/*δ* agonist GW501516 [41]. Moreover, activation of PPAR*β*/*δ* upregulated VEGF in colon carcinoma cells, promoting colon tumour epithelial cell survival through activation of AKT signalling [41]. Angiogenesis was not studied in this model, however, any substantial tumour growth requires a blood supply and angiogenesis to allow it to develop. In contrast, in human colon and liver cancer cell lines [40], PPAR*β*/*δ* ligands had no effect on human cancer cell growth, AKT, VEGF or COX-2 expression in vitro or on these makers in the liver, colon, and colon polyps in mice treated in vivo [40]. The roles of PPAR*β*/*δ* in VEGF- mediated tumorigenesis are, therefore, still in need of further clarification.

### 3.4. Expression of PPAR*β*/*δ* in the eye

Angiogenesis regulates both the physiological development and many of the most common pathophysiology’s of the eye. As yet, there is no direct evidence linking PPAR*β*/*δ* and angiogenesis in the eye, however, PPAR*β*/*δ* is clearly expressed at least in murine ocular tissue. PPAR*β*/*δ* is expressed in the eye ciliary body epithelial cells, cornea epithelial cells, cornea endothelium, cornea fibroblast, retina inner nuclear layer, and retina ganglion cell layer [42]. Although one must be cautious interpreting data from nonocular tissue to the eye [43], as discussed previously and following, pathways that have direct relevance to ocular angiogenesis are clearly regulated by PPAR*β*/*δ* and are therefore worthy of discussion.

## 4. VEGF AND OCULAR ANGIOGENESIS

VEGF is essential in retinal vasculature development [44]. Initially blood vessels grow from the optic nerve outwards. As the retinal tissue develops via a complex interplay between different cellular components such as neurons, glia, endothelial cells, pericytes, and immune cells, the increased oxygen demand induces hypoxia, the main stimulant for new vessel growth via angiogenesis. As the tissue/vasculature develops and gets oxygenated, hypoxia and VEGF decrease limiting new vessel growth [44].

In contrast, neovascularisation of the adult eye via angiogenesis is a critical component of many disorders of the eye including retinopathy of prematurity, diabetic retinopathy, and age-related macular degeneration, the latter two being the leading causes of blindness in the Western world (as reviewed in detail elsewhere [29, 45–48]). Pathological neovascularisation resulting from tissue damage and hypoxia results in a more complex “inflammatory” angiogenesis. These new vessels are often fragile and leaky leading to haemorrhage and vision disturbance and loss. The main trigger for this new vessel growth still appears to be hypoxia induced VEGF expression [29, 45–48]. Angiogenesis is a homeostatic repair mechanism that is required for the reoxygenation of the damaged ischemic tissue [29, 45–48]. The problems that arise with pathologies such as age-related macular degeneration and diabetic retinopathy are that this new vessel growth is leaky and has a critical inflammatory component. VEGF (in particular VEGF A; VEGF_165_) in addition to directly stimulating angiogenesis is also a potent vascular permeability factor and appears to play a role in regulating the local inflammation associated with pathological neovascularisation [49]. VEGF has become a clear therapeutic target for the treatment of angiogenesis in the eye. The clinical importance of VEGF as a target has recently been further demonstrated with the development and use of two new drugs targeting its actions: Macugen (pegaptanib), an aptamer, and Lucentis (ranibizumab), a FAB fragment, from a humanised monoclonal antibody, which both functionally block VEGF. Moreover, Macugen and Lucentis both show clinical efficacy in patients with age-related macular degeneration [50]; especially when treated early and a mature neovasculature has yet to form. These therapies require local delivery by intravitriol injection, which although having the benefit of overcoming problems such as systemic VEGF blockade, they are clearly still not ideal, and show that new therapies are still required.

## 5. PPAR*β*/*δ* OCULAR ANGIOGENESIS, INFLAMMATION, AND COAGULATION

Angiogenesis associated with pathophysiology is often associated with multiple process such as tissue damage, inflammation, and coagulation. In contrast, developmental angiogenesis may be a simpler hypoxia driven event. Indeed, an inflammatory response is induced by VEGF during pathological but not physiological ischemia-induced retinal angiogenesis [51, 52]. Moreover, specifically blocking inflammatory cytokines monocyte chemotactic protein-1 and macrophage inflammatory protein-1a can reduce retinal neovascularisation [53]. Tissue factor is a critical initiator of blood coagulation, and is associated with tumour aggressiveness and angiogenesis in a variety of cancer cells [54], as well as in choroidal neovascularisation where it promotes fibrin formation and the growth of the choroidal angiogenic complex [55]. One important facet of pathological angiogenesis may therefore be this involvement additional pathways, and a complex interplay between processes of tissue damage, hypoxia, inflammation, and coagulation. A long-term therapeutic aim may therefore be to have revascularisation of hypoxic tissue similar to development without these additional inflammatory/coagulation processes.

PPAR*β*/*δ* induces VEGF in a number of cell types and induces angiogenesis. Therefore, one may predict that a PPAR*β*/*δ* antagonist would be useful to treat or at least test in models of eye disease that involve neovascularisation. However, PPAR*β*/*δ* seems consistent with other PPAR's in that it also has anti-inflammatory and anticoagulation properties, suggesting that its properties in ocular angiogenesis may be more complex than one would originally predict.

PPAR*β*/*δ* activation suppresses endothelial cell tissue factor expression [12]. PPAR*β*/*δ* is also expressed in platelets where its ligands reduce platelet aggregation to a variety of stimuli [56]. Similar to PPAR*α* and PPAR*γ*, PPAR*β*/*δ* ligands are anti-inflammatory in endothelial cells, inhibiting TNF*α*-induced upregulation of expression of vascular cell adhesion molecule-1, monocyte chemoattractanct protein-1, and nuclear factor (NF)*κ*B translocation [57]. In macrophages, PPAR*β*/*δ* controls inflammatory status by its association and disassociation with the transcriptional repressor BCL-6 [58]; in the absence of ligand, PPAR*β*/*δ* physically associates with and inhibits this anti-inflammatory BCL-6. When a PPAR*β*/*δ* ligand is added, BCL-6 dissociates from PPAR*β*/*δ* and represses the inflammation and levels of monocyte chemoattractanct protein-1, -3, and IL-1*β* [58].

## 6. CONCLUSION

PPAR*β*/*δ* induces angiogenesis and protects endothelial cells from oxidant damage. A common signal for PPAR*β*/*δ* activation in endothelial cells or surrounding tissue may be the induction of VEGF. PPAR*β*/*δ* is expressed in all tissues in the eye, however its function has yet to be tested in physiological processes, development, or pathophysiological disorders. The development of both the eye and common pathological disorders requires angiogenesis, with VEGF being a primary signalling molecule. Blocking PPAR*β*/*δ* may therefore provide a new therapy to treat angiogenic eye disorders. The difference between “physiological” and “pathophysiological” angiogenesis may be additional components of inflammation and coagulation. PPAR*β*/*δ* ligands reduce inflammation and components of the coagulation cascade. It will be of great interest to test the roles of PPAR*β*/*δ* in the eye as a potential proangiogenic stimulus reliving the hypoxia, while potentially still capable of reducing the damaging inflammatory/coagulation signals.

## Figures and Tables

**Figure 1:**
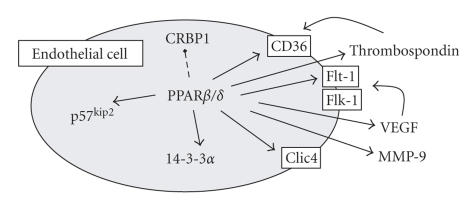
Proangiogenic/prosurvival pathways of PPAR*β*/*δ* in endothelial cells. PPAR*β*/*δ* is expressed in endothelial cells. PPAR*β*/*δ* activation induces (solid line) the expression of VEGF and its receptor Flt-1, matrix metalloproteinase (MMP)-9, thrombospondin and its receptor CD36, the chloride intracellular channel protein (CLIC)-4, the cell cycle inhibitor p57^kip2^, and the antiapoptotic protein 14-3-3*α*. In contrast, the cellular retinol binding protein-1 is decreased (dashed line) by PPAR*β*/*δ* activation. For those interested, a complex transcriptional map of the potential role of PPAR*β*/*δ* as a hub node in tumour angiogenesis has recently also been formed as detailed in [32].

**Figure 2:**
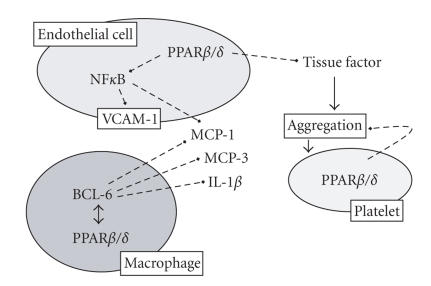
Anti-inflammatory/anticoagulation pathways of PPAR*β*/*δ*. PPAR*β*/*δ* activation in endothelial cells reduces NF*κ*B activation and the induction of vascular cell adhesion molecule (VCAM)-1, and monocyte chemoattractant protein (MCP)-1, along with the release of tissue factor. PPAR*β*/*δ* is expressed in platelets and monocytes/macrophages. PPAR*β*/*δ* ligands reduce platelet aggregation via a rapid nongenomic mechanism. In macrophages, PPAR*β*/*δ* ligands release the transcriptional corepressor BCL-6 from its complex with PPAR*β*/*δ*. Free BCL-6 suppresses the release of MCP-1, MCP-3, and IL-1*β*.
